# Thioredoxin-1 Protects Spinal Cord from Demyelination Induced by Methamphetamine through Suppressing Endoplasmic Reticulum Stress and Inflammation

**DOI:** 10.3389/fneur.2018.00049

**Published:** 2018-02-06

**Authors:** Lihua Yang, Yinli Guo, Mengbin Huang, Xiaoli Wu, Xiang Li, Guobing Chen, Ye Li, Jie Bai

**Affiliations:** ^1^Faculty of Environmental Science and Engineering, Kunming University of Science and Technology, Kunming, China; ^2^Medical School, Kunming University of Science and Technology, Kunming, China; ^3^Narcotics Control School, Yunnan Police College, Kunming, China

**Keywords:** methamphetamine, demyelination, thioredoxin-1, endoplasmic reticulum stress, inflammation, spinal cord

## Abstract

Methamphetamine (METH) is a psychostimulant abused around the world. Emerging evidence indicates that METH causes brain damage. However, there are very few reports on METH-induced demyelination. Thioredoxin-1 (Trx-1) is a redox regulating protein and plays the roles in protecting neurons from various stresses. However, whether Trx-1 resists demyelination induced by METH has not been reported. In this study, we found that METH-induced thin myelin sheaths in spinal cord, whereas Trx-1 overexpression transgenic (TG) mice restored the myelin sheaths thickness. The expressions of myelin-associated glycoprotein, myelin basic protein, and cyclin-dependent kinase 5 were decreased by METH, whereas these alterations were blocked in Trx-1 TG mice. The expressions of procaspase-12 and procaspase-3 were decreased by METH, the expression of calpain1 was increased by METH, whereas the alterations were suppressed in Trx-1 TG mice. As same as, the expressions of the extracellular signal-regulated kinase, nuclear factor κB, tumor necrosis factor-alpha, and interleukin-1beta were induced by METH, which were suppressed in Trx-1 TG mice. These data suggest that Trx-1 may play a critical role in resisting the METH-mediated demyelination in spinal cord through regulating endoplasmic reticulum stress and inflammation pathways.

## Introduction

Methamphetamine (METH) is widely abused in the world and leads to increasing positive mood and euphoria (1, 2). The brain is the primary focus of most studies discovering neural mechanisms on addictive drugs (3). The inseparable part of the central nervous system (CNS), spinal cord results from multiple pathologies due to its function of connecting the brain with the body. However, spinal cord injury (SCI) induced by addictive drugs attracts less attention. It has been reported that opioid addiction affects myelination (4). It is still unknown whether METH induces demyelination in spinal cord.

Demyelination is one of the most important pathological factors of SCI, and it is an acquired disorder in which normally formed myelin degenerates after exposing axons to the extracellular environment. Oxidative stress (5), inflammation (6), endoplasmic reticulum (ER) stress (7), ecotoxicity, and dysregulation of metabolic processes are involved in SCI. After traumatic SCI, ER stress exacerbates secondary injury, leads to expansion of demyelination and reduction of remyelination resulting from oligodendrocyte precursor cell apoptosis (4).

Thioredoxin-1 (Trx-1) is a redox regulating protein with the redox-active cysteine residues in its active site sequence: –Cys–Gly–Pro–Cys–. The cellular activity of Trx-1 is regulated by its total expression, localization (nucleus or cytosol), protein–protein interactions, and posttranslational modification (8). In the extracellular environment, Trx-1 exhibits chemokine-like activity (9) and in the cytoplasm scavenges reactive oxygen species, radical and hydrogen peroxide, and activates transcriptional factors. Trx-1 is involved in cell proliferation, apoptosis, and neuron protection (10–12). Our study has shown that Trx-1 protects PC12 cells from METH-induced toxicity (13). Trx-1 protects neurons from damage by suppressing ER stress (14). However, whether Trx-1 resists demyelination in spinal cord induced by METH has not been reported.

In this study, we suppose that Trx-1 could resist demyelination in spinal cord induced by METH. We examined myelin thickness of spinal cord, the expressions of myelin-associated glycoprotein (MAG), myelin basic protein (MBP), cyclin-dependent kinase 5 (CDK5), ER stress, and inflammatory factors. Our results suggest that Trx-1 may play a role in resisting METH-induced demyelination in spinal cord.

## Materials and Methods

### Materials

Methamphetamine was obtained from Yunnan Province Public Security Department and dissolved in sterile water. Anti-mouse Trx-1 rabbit polyclonal antibody (14999-1-AP; 1:1,000) was purchased from ProteinTech (Wuhan, China). The antibodies MAG (sc-15324; 1:1,000), MBP (sc-376995; 1:1,000), procaspase-12 (sc-5627, 1:1,000), calpain1 (sc-13990; 1:1,000), phosphorylation extracellular signal-regulated kinase (p-ERK, sc-7383; 1:1,000), extracellular signal-regulated kinase (ERK, sc-94; 1:1,000), and β-actin (sc-47778; 1:1,000) were purchased from Santa Cruz Bio Technology (Santa Cruz, CA, USA). The antibodies procaspase-3 (19677-1-AP, 1:2,000) and nuclear factor κB (NF-κB) (10745-1-AP, 1:2,000) were purchased from Proteintech Group (Proteintech Group, Inc., Wuhan, Hubei, China). The antibody CDK5 (Ab-40773; 1:2,000) was purchased from Abcam (Abcam plc, Cambridge, UK). qRT-PCR primers [β-actin, interleukin-1beta (IL-1β), and tumor necrosis factor-alpha (TNF-α)] were purchased from Sangon Biotech Co., Ltd. (Shanghai, China).

### Animals

Male C57BL/6 mice (Chongqing Medical University, China) and human Trx-1 transgenic (TG) mice, 7–8 weeks of age, were used in the experiments. The mice were housed in plastic cages and maintained on a 12 h light–dark cycle and had free access to food and water. C57BL/6 human TG mice were constructed by (Cyagen Biosciences Inc., Guangzhou, China). The pronuclei of fertilized eggs from hyper ovulated C57BL/6 were microinjected with hTrx-1 cDNA construct. The presence of Trx-1 also was confirmed by reverse transcription-PCR analysis. Mice were divided into four groups, control group, METH group, TG group, and TG + METH group (each group *n* = 9). All protocols and procedures are approved by the animal ethics council of Kunming University of Science and Technology and are in accordance with the National Institutes of Health Guide to the Care and Use of Laboratory Animals and are approved by the local Committee on Animal Use and Protection of Yunnan province (No. LA2008305).

### Drug Treatments

**Figure 1:**
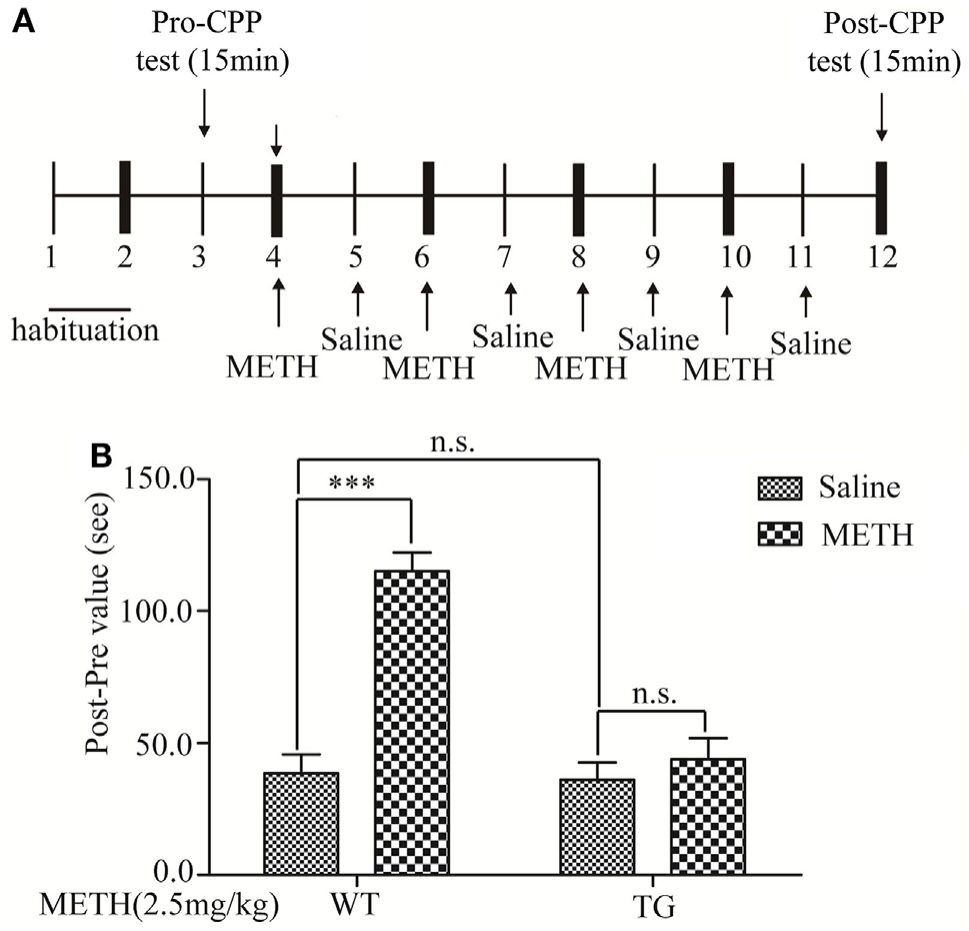
Effects of thioredoxin-1 (Trx-1) overexpression on methamphetamine (METH)-induced conditioned place preference paradigm (CPP). **(A)** Experimental schedule for measurement of METH-induced CPP in mice. **(B)** Trx-1 attenuated METH-induced CPP. Values are means ± SE (*N* = 9); ***P < 0.001.

Mice (25–30 g) were administered with METH (2.5 mg/kg) and saline solution by intraperitoneal injections (IP) for 8 days, METH and saline injection interval for 24 h. Control group mice were injected saline 8 days (Figure 1A). Mice were sacrificed after the behavioral test, and the thoracic spinal cord was quickly dissected out, frozen, and stored in a deep freezer at −80°C until the assays.

### Conditioned Place Preference

The apparatus used for the place-conditioning task consisted of two compartments: a black Plexiglas box and a white Plexiglas box (15 cm × 15 cm × 30 cm). To enable the mice to distinguish easily the white box from the black one, the floors of the white and black boxes were covered with mesh and frost plastic plexiglas, respectively. Each box could be divided by a sliding door. The experimental schedule for the conditioned place preference task is shown in (Figure 1A). Mice were placed into the box and allowed to move freely between the white and the black boxes for 15 min once per day for 3 days (days 1–3) for the precondition. On day 3, the time that the mouse spent in each box was measured as precondition. On days 4, 6, 8, and 10, mice were treated with METH and confined in either the white or the black box for 15 min. On days 5, 7, 9, and 11, mice were given saline and placed opposite to the METH-conditioning box (black box) for 15 min. On day 12, the post-conditioning test was performed without drug treatment, and the time the mice spent in each box was measured for 15 min.

### Electron Microscopy

For ultrastructural analyses, mice were perfused with 4% paraformaldehyde, 2% glutaraldehyde in 0.1 M cacodylate buffer, pH 7.4 (Electron Microscopy Sciences). Thoracic spinal cords were post-fixed in 1% OsO_4_. Samples were dehydrated through graded ethanol, stained en bloc with uranyl acetate, and embedded in a Poly/Bed812 resin (Polysciences Inc., Warrington, PA, USA). Thin (1 µm) sections were stained with toluidine blue, and ultrathin (0.1 µm) sections from matching areas of experimental and control tissue blocks were cut and visualized using an electron microscope (JEOL1200CX) at 80 kV. Between 100 and 400 axons were measured per genotype from matched regions of the thoracic spinal cord. For g-ratios, non-overlapping digitized images of fiber cross-sections from dorsal roots were obtained and analyzed using image pro plus software. g-Ratios were calculated by dividing the axon diameter by the total fiber diameter. g-Ratios were determined exclusively in the dorsal root due to their mix of small- and large-caliber axons.

### Western Blot

Protein lysates were prepared by using the solubilizing solution [20 mM Tris–HCl (pH 7.4), 150 mM NaCl, 1% NP-40, 1 mM EDTA, 1 mM PMSF, 1 mM EGTA, 1% Triton X-100, 2.5 mM sodium pyrophosphate, 1 mM Na_3_VO_4_, 1 mM β-glycerolphosphate, and 1 mg/ml leupeptin]. Protein concentration was determined using Bio-Rad protein assay reagent (Hercules, CA, USA). An equal quantity of proteins was separated by 12 or 15% SDS-PAGE and transferred to a PVDF membrane (Millipore Corporation, Billerica, MA, USA). The membrane was soaked in 10% skim milk (in PBS, pH 7.2, containing 0.1% Tween-20) overnight at 4°C, then incubated with primary antibodies followed by peroxidase-conjugated anti-mouse or anti-rabbit IgG (KPL, Inc., Gaithersburg, MD, USA). The epitope was visualized by an ECL Western blot detection kit (Millipore Corporation, Billerica, MA, USA). Densitometry analysis was performed by using ImageJ software.

### RNA Extraction and Quantitative PCR Analysis

Total RNA was extracted from 0.1 g spinal cord tissue by using a Trizol reagent kit (CWBIO Corporation, Beijing, China) and converted to cDNA by using the Revert Aid TM First Strand cDNA Synthesis Kit (Fermentas, Walldorf Baden, Germany). The product was analyzed by using a Prism 7300 Sequence Detection System (Applied Biosystems, Foster, CA, USA). The following primer pairs were selected for real-time polymerase chain reaction: gene expression was calculated relative to the housekeeping gene mouse β-actin F: 5′-CAG TTC GCC ATG GAT GAC GAT-3′, R: 5′-ATC TGG GTC ATC TTT TCA CGG TTG-3′; mouse TNF-α F: 5′-GCC TAT GTC TCA GCC TCT TCT C-3′, R: 5′-TGG GAA CTT CTC ATC CCT TTG G-3′; and mouse IL-1β F: 5′-TGC CAC CTT TTG ACA GTG ATG-3′, R: 5′-TGA TGT GCT GCT GCG AGA TT-3′.

### Statistical Analysis

Data were expressed as mean ± SD values. Statistical analysis was performed using GraphPad Prism 5 software. The two-way ANOVA followed by a *post hoc* multiple comparison test was used to compare control and treatment groups. *P* values of less than 0.05 were considered statistically significant. All blots are representative of experiments that were performed at least more than 6.

## Results

### The Conditioned Place Preference Was Induced by METH

Associative learning between contextual cues and the rewarding effects of abused substances can result in CPP, a behavior observed in rodents (15). In this study, we determined METH-induced rewarding effect by using CPP in control group mice. The experimental schedule was described in Figure 1A. The result showed that the CPP was blocked in TG mice (Figure 1B). Two-way ANOVA showed a significant mice × drug interaction (*F*_1, 20_ = 22.59, *P* < 0.001) and significant influence of drug (*F*_1, 20_ = 34.07, *P* < 0.001) and mice (*F*_1, 20_ = 26.00, *P* < 0.001). Bonferroni *post hoc* test revealed significant difference between the control and METH group (*P* < 0.001), but no significant difference in TG mice and TG mice + METH group (*P* > 0.05).

### Overexpression of Trx-1 Blocked Trx-1 Decrease Induced by METH in Spinal Cord

**Figure 2:**
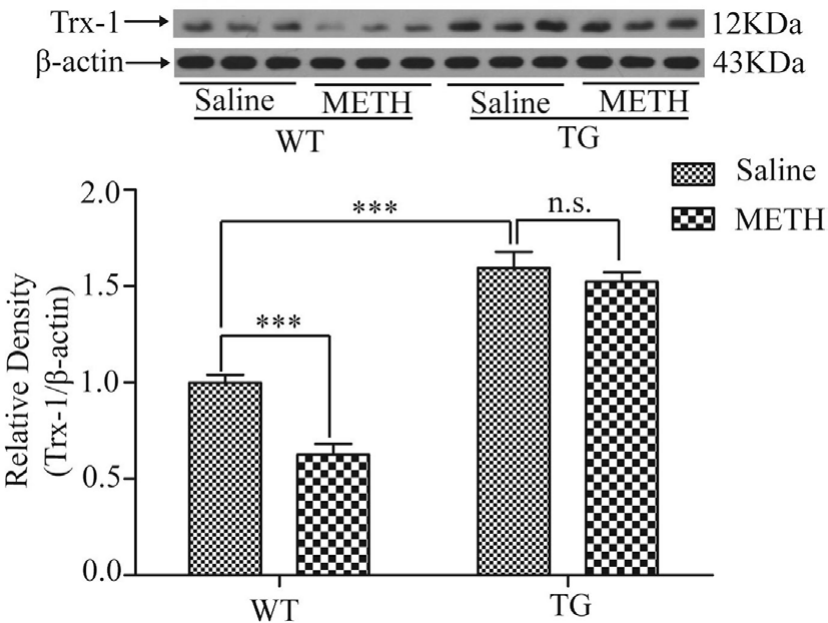
Overexpression of thioredoxin-1 (Trx-1) prevented decrease of Trx-1 induced by methamphetamine (METH) in spinal cord. Values are means ± SE (*N* = 6); ***P < 0.001.

To investigate whether Trx-1 is regulated by METH in spinal cord, the expression of Trx-1 was examined after METH treatment by Western blot. The expression of Trx-1 was decreased by METH, which was restored in TG mice (Figure 2). Two-way ANOVA showed a significant mice × drug interaction (*F*_1, 20_ = 6.36, *P* < 0.05) and significant influence of drug (*F*_1, 20_ = 13.93, *P* < 0.01) and mice (*F*_1, 20_ = 1, *P* < 0.001). Bonferroni *post hoc* test revealed significant difference between the control and METH group (*P* < 0.001), but no significant difference in TG group and TG + METH group by METH (*P* > 0.05). The *post hoc* test also revealed significant difference between the TG group and control group (*P* < 0.001).

### Overexpression of Trx-1 Restored Decrease of Myelin Thickness Induced by METH in Spinal Cord

**Figure 3:**
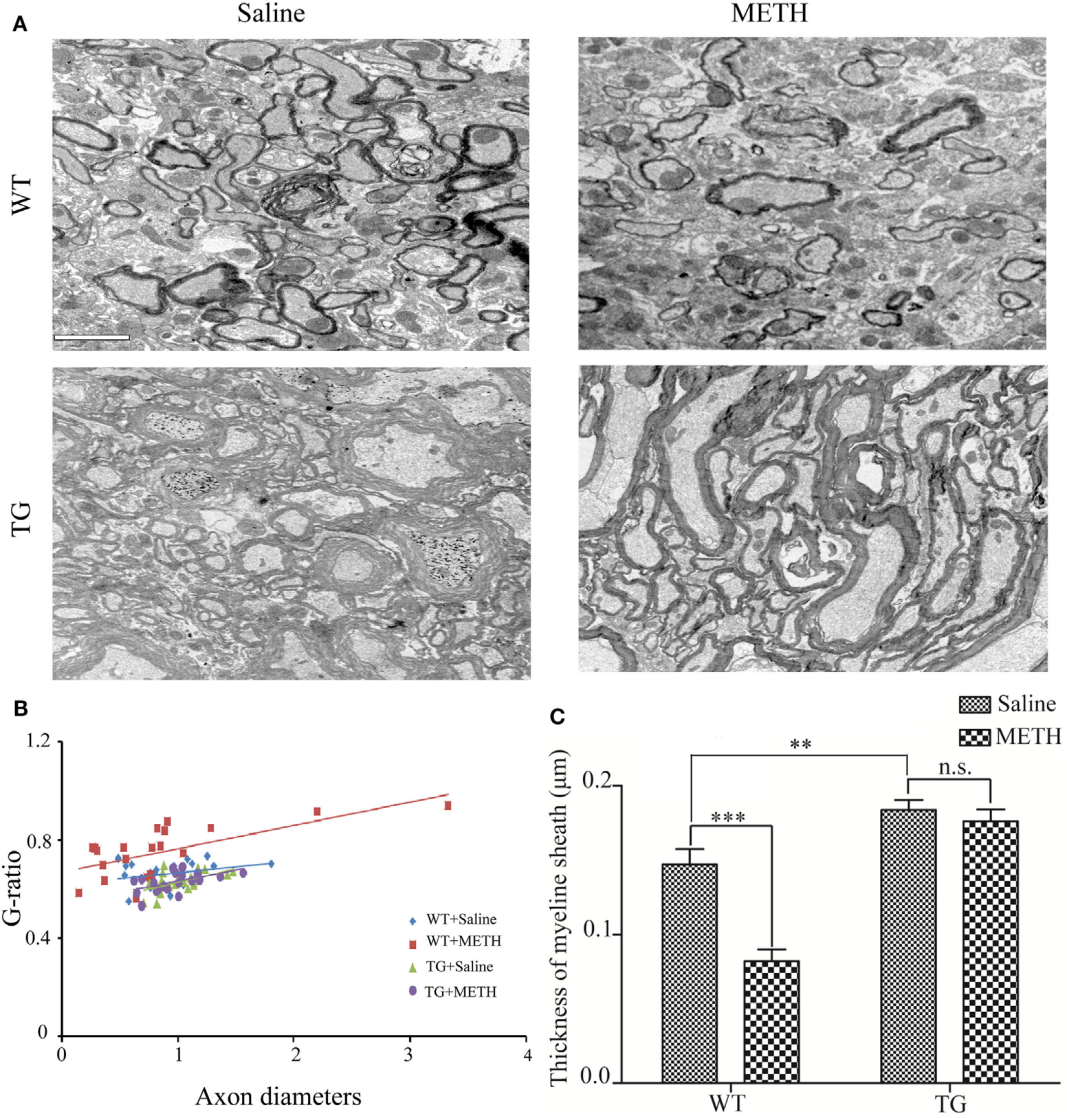
Overexpression of thioredoxin-1 (Trx-1) prevented decrease of myelin thickness induced by methamphetamine (METH) in spinal cord. **(A)** EM images show overexpression of Trx-1 prevented decrease of myelin thickness induced by METH in spinal cord average myelin thickness: control mice 0.147 ± 0.007; METH mice 0.082 ± 0.008; TG mice 0.183 ± 0.004; and TG mice by METH 0.179 ± 0.008. **(B)** A significant increase in the g-ratio in mice by METH. Overexpression of Trx-1 suppressed increase of g-ratio by METH in spinal cord. **(C)** Overexpression of Trx-1 prevented decrease of myelin thickness induced by METH in spinal cord. Values are means ± SE (*N* = 6); **P < 0.01, and ***P < 0.001.

To determine the effect of METH on myelin thickness, the thoracic spinal cord section was examined by electron microscopy. The result showed that myelin sheaths of axons were decreased by METH, which were restored in TG mice (Figure 3A). A significant increase in the g-ratio indicates very thin myelin sheaths in METH mice, which was inhibited in Trx-1 TG mice by METH (Figure 3B). Two-way ANOVA showed a significant mice × drug interaction (*F*_1, 20_ = 9.31, *P* < 0.01) and significant difference of drug (*F*_1, 20_ = 10.29, *P* < 0.01) and mice (*F*_1, 20_ = 33.37, *P* < 0.001). Bonferroni *post hoc* test revealed significant difference between the control and METH group (*P* < 0.001), but no significant difference between TG mice and TG + METH mice (*P* > 0.05). The *post hoc* test also revealed significant difference between the TG and control mice (*P* < 0.001). Morphometric quantification of average myelin thickness confirmed a relative reduction of myelin thickness treated by METH compared with control mice. However, the decrease of myelin thickness was blocked in TG mice (Figure 3C). Two-way ANOVA showed a significant mice × drug interaction (*F*_1, 20_ = 12.11, *P* < 0.001) and significant influence of drug (*F*_1, 20_ = 19.35, *P* < 0.001) and mice (*F*_1, 20_ = 62.04, *P* < 0.001). Bonferroni *post hoc* test revealed significant difference between the control and METH group (*P* < 0.001), but no significant difference between TG mice and TG + METH mice (*P* > 0.05). The *post hoc* test also revealed significant difference between the TG and control mice (*P* < 0.01).

### Overexpression of Trx-1 Restored the Expressions of Myelin Proteins Induced by METH in Spinal Cord

**Figure 4:**
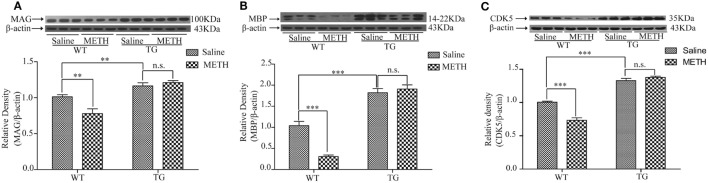
Overexpression of thioredoxin-1 (Trx-1) regulated the expressions of major myelin proteins by methamphetamine (METH) in spinal cord. **(A)** Trx-1 overexpression inhibited decrease of myelin-associated glycoprotein (MAG) induced by METH in spinal cord. **(B)** Trx-1 overexpression prevented decrease of myelin basic protein (MBP) induced by METH in spinal cord. **(C)** Trx-1 overexpression restored decrease of cyclin-dependent kinase 5 (CDK5) induced by METH in spinal cord. Values are means ± SE (*N* = 6); **P < 0.01, and ***P < 0.001.

MAG and MBP are mainly located in myelin, which play the important role in axon–myelin integrity (8, 9). Then, we examined MAG expression of spinal cord after METH administration. The results showed that the MAG expression was decreased by METH, which was restored in TG mice (Figure 4A). Two-way ANOVA showed a significant mice × drug interaction (*F*_1, 20_ = 10.08, *P* < 0.01) and significant difference of drug (*F*_1, 20_ = 4.52, *P* < 0.05) and mice (*F*_1, 20_ = 3.65, *P* < 0.001). Bonferroni *post hoc* test revealed significant difference between the control and METH group (*P* < 0.01), but no significant difference between TG mice and TG + METH mice (*P* > 0.05). The *post hoc* test also revealed significant difference between the TG and control mice (*P* < 0.01). The expression of MBP was decreased by METH, which was restored in TG mice (Figure 4B). Two-way ANOVA showed a significant mice × drug interaction (*F*_1, 20_ = 22.01, *P* < 0.001) and significant difference of drug (*F*_1, 20_ = 13.68, *P* < 0.01) and mice (*F*_1, 20_ = 185.76, *P* < 0.001). Bonferroni *post hoc* test revealed significant difference between the control and METH group in wild-type (WT) mice (*P* < 0.001), but no significant difference between TG mice and TG + METH mice (*P* > 0.05). The *post hoc* test also revealed significant difference between the TG and control mice (*P* < 0.001).

### Overexpression of Trx-1 Restored the Expression of CDK5 Inhibited by METH in Spinal Cord

CDK5 plays a role in oligodendrocyte development and myelination. So, we examined the expression of CDK5 in spinal cord after METH treatment. The results showed that the CDK5 expression was decreased by METH, which was restored in TG mice (Figure 4C). Two-way ANOVA showed a significant mice × drug interaction (*F*_1, 20_ = 32.45, *P* < 0.001) and significant difference of drug (*F*_1, 20_ = 15.79, *P* < 0.001) and mice (*F*_1, 20_ = 294.26, *P* < 0.001). Bonferroni *post hoc* test revealed significant difference between the control and METH group in WT mice (*P* < 0.001), but no significant difference in TG mice and TG + METH mice (*P* > 0.05). The *post hoc* test also revealed significant difference between the TG and control mice (*P* < 0.001).

### Overexpression of Trx-1 Inhibited METH-Induced Demyelination through Regulating ER-Mediated Apoptosis in Spinal Cord

**Figure 5:**
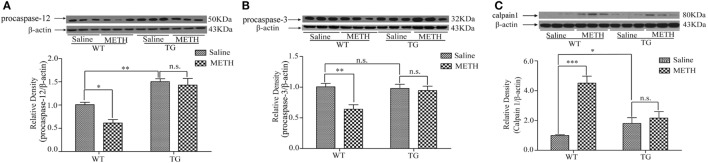
Overexpression of thioredoxin-1 (Trx-1) inhibited endoplasmic reticulum stress induced by methamphetamine (METH) in spinal cord. **(A)** Overexpression of Trx-1 prevented decrease of procaspase-12 induced by METH in spinal cord. **(B)** Overexpression of Trx-1 prevented decrease of procaspase-3 induced by METH in spinal cord. **(C)** Overexpression of Trx-1 suppressed increase of calpain1 induced by METH in spinal cord. Values are means ± SE (*N* = 6); *P < 0.05, **P < 0.01, and ***P < 0.001.

Caspase-12 is specific for ER stress-induced apoptosis (10), caspase-3 is the downstream of caspase-12. Thus, we examined the expressions of procaspase-12 and procaspase-3. The results showed that the expression of procaspase-12 was decreased by METH, which was restored in TG mice (Figure 5A). Two-way ANOVA showed a significant mice × drug interaction (*F*_1, 20_ = 4.57, *P* < 0.05) and significant influence of drug (*F*_1, 20_ = 6.77, *P* < 0.05) and mice (*F*_1, 20_ = 63.19, *P* < 0.001). Bonferroni *post hoc* test revealed significant difference between the control and METH group in WT mice (*P* < 0.01), but no significant difference between TG mice and TG mice by METH (*P* > 0.05). The *post hoc* test also revealed significant difference between the TG and control mice (*P* < 0.01). The expression of procaspase-3 was decreased by METH, which was restored in TG mice (Figure 5B). Two-way ANOVA showed a significant mice × drug interaction (*F*_1, 20_ = 9.52, *P* < 0.01) and significant difference of drug (*F*_1, 20_ = 6.74, *P* < 0.05) and mice (*F*_1, 20_ = 4.62, *P* < 0.05). Bonferroni *post hoc* test revealed significant difference between the control and METH group (*P* < 0.01), but no significant difference between TG mice and TG + METH mice (*P* > 0.05). The expression of calpain1 in TG mice was significantly higher than in control mice, and Trx-1 overexpression inhibited the further increase of calpain1 induced by METH (Figure 5C). Two-way ANOVA showed a significant mice × drug interaction (*F*_1, 20_ = 24.33, *P* < 0.001) and significant difference of drug (*F*_1, 20_ = 30.30, *P* < 0.001) and mice (*F*_1, 20_ = 6.42, *P* < 0.05). Bonferroni *post hoc* test revealed significant difference between the control and METH group (*P* < 0.001), but no significant difference between TG mice and TG + METH mice (*P* > 0.05).

### Overexpression of Trx-1 Inhibited p-ERK Induced by METH in Spinal Cord

**Figure 6:**
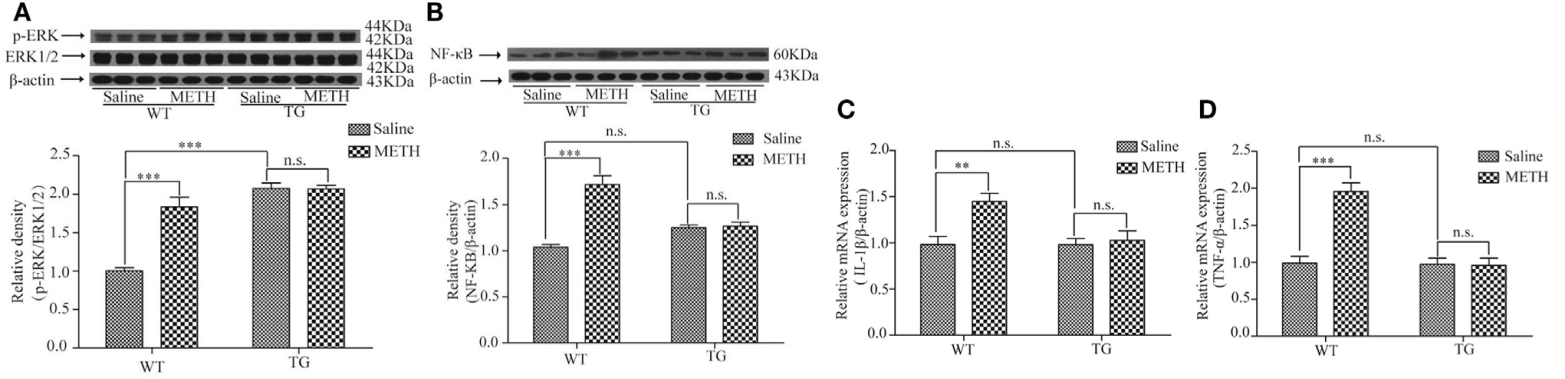
Overexpression of thioredoxin-1 (Trx-1) suppressed the expressions of phosphorylation extracellular signal-regulated kinase (p-ERK) and nuclear factor κB (NF-κB) and suppressed the levels of tumor necrosis factor-alpha (TNF-α) and interleukin-1beta (IL-1β) mRNA induced by methamphetamine (METH) in spinal cord. **(A)** Overexpression of Trx-1 suppressed the expression of p-ERK induced by METH in spinal cord. **(B)**Trx-1 overexpression decreased the expression of NF-κB induced by METH in spinal cord. **(C)** Trx-1 overexpression decreased the level of IL-1β mRNA induced by METH in spinal cord. **(D)** Trx-1 overexpression decreased the level of TNF-α mRNA induced by METH in spinal cord. Values are means ± SE (*N* = 6); **P < 0.01, and ***P < 0.001.

The mitogen-activated protein kinase (MAPK) signaling pathway, ERK–c-Jun N-terminal kinase (JNK)–p38 MAPKs, is involved in the regulation of pro-inflammatory cytokines (16). Studies have confirmed that ERK can be activated by METH (17, 18). We investigated whether ERK and p-ERK were induced by METH in spinal cord, as we expected, the activity of p-ERK was increased after METH treatment, whereas the activity of ERK was not further increased in Trx-1 TG mice (Figure 6A). Two-way ANOVA showed a significant mice × drug interaction (*F*_1, 20_ = 29.36, *P* < 0.001) and significant influence of drug (*F*_1, 20_ = 28.48, *P* < 0.001) and mice (*F*_1, 20_ = 71.20, *P* < 0.001). Bonferroni *post hoc* test revealed significant difference between the control and METH group (*P* < 0.001), but no significant difference between TG mice and TG + METH mice (*P* > 0.05).

### Overexpression of Trx-1 Suppressed the METH-Induced Inflammation in Spinal Cord

The inflammation is usually involved in demyelination (19). So, we further examined that whether the expressions of NF-κB, IL-1β, and TNF-α were induced by METH in spinal cord. Our result showed that the expression of NF-κB was increased by METH, which was suppressed in TG mice (Figure 6B). Two-way ANOVA showed a significant mice × drug interaction (*F*_1, 20_ = 35.16, *P* < 0.001) and significant difference of drug (*F*_1, 20_ = 38.78, *P* < 0.001) and mice (*F*_1, 20_ = 4.51, *P* < 0.05). Bonferroni *post hoc* test revealed significant difference between the control and METH group (*P* < 0.001), but no significant difference between TG mice and TG + METH mice (*P* > 0.05). The results also showed that the mRNA level of IL-1β was increased by METH, which was inhibited in TG mice (Figure 6C). Two-way ANOVA showed a significant mice × drug interaction (*F*_1, 20_ = 5.76, *P* < 0.05) and significant difference of drug (*F*_1, 20_ = 8.70, *P* < 0.01) and mice (*F*_1, 20_ = 5.90, *P* < 0.05). Bonferroni *post hoc* test revealed significant difference between the control and METH group (*P* < 0.01), but no significant difference between TG mice and TG + METH mice (*P* > 0.05). The mRNA level of TNF-α was increased by METH, which was suppressed in TG mice (Figure 6D). Two-way ANOVA showed a significant mice × drug interaction (*F*_1, 20_ = 24.85, *P* < 0.001) and significant difference of drug (*F*_1, 20_ = 23.08, *P* < 0.001) and mice (*F*_1, 20_ = 26.32, *P* < 0.001). Bonferroni *post hoc* test revealed significant difference between the control and METH group (*P* < 0.001), but no significant difference between TG mice and TG + METH mice (*P* > 0.05).

## Discussion

In this study, we found that myelin thickness was decreased, expressions of MAG, MBP and CDK5 were decreased after METH treatment, these alterations were suppressed in TG mice. The declined expressions of procaspase-12 and procaspase-3 in spinal cord after METH treatment were blocked in TG mice, and the elevated expressions of calpain1 and NF-κB, the levels of IL-1β and TNF-α mRNA in spinal cord after METH treatment were inhibited in TG mice.

Conditioned place preference paradigm is a model for assessing rewarding effect. Our previous study certified that Trx-1 overexpression occluded the CPP expression induced by METH. There was no significant change in locomotor activity between control group and METH group after METH treatment (20). So, we concluded that METH did not cause acute movement disorder from SCI in mice.

The demyelination appears the decrease of myelin thickness and decrease expressions of MAG and MBP. Thus, we examined the myelin thickness after METH treatment. Our result confirmed that the diameter of axon which was wrapped by myelin was significantly thinner in METH group than in control (Figures 3A–C) by the quantification for myelin thickness of g-ratio analysis. Myelin is important for axon maintenance and function, demyelination results in damage or loss of the myelin sheath around axons (21). Our results showed that METH treatment induced demyelination in spinal cord, whereas overexpression of Trx-1 prevented decrease of myelin thickness induced by METH in spinal cord. Thus, Trx-1 may protect spinal cord from demyelination induced by METH treatment.

MAG is known to enrich the periaxonal layers of the myelin sheath, thus it is myelin-specific proteins (22). MBP is essential for the compaction and stability of specific oligodendrocyte membranes that concentrically wrapped around neurons in multilamellar arrangements, forming the myelin sheath (23). Our results showed that MAG and MBP expressions were decreased by METH in group mice, suggesting that METH treatment induces demyelination in spinal cord. Interestingly, the expressions of MAG and MBP in TG mice were significantly higher than in control mice. Thus, the decreased expressions of MAG and MBP were restored in TG mice (Figures 4A,B). Thus, our results suggest that the increased expressions of MAG and MBP in TG mice may protect spinal cord from demyelination induced by METH treatment.

Multiple signals influence the rate and extent of CNS myelination. CDK5 is a serine/threonine kinase with significant homology to cell cycle related CDKs. Recently, oligodendrocyte development and myelination have been shown to be influenced by CDK5 (24–26). Localized pharmacological inhibition of CDK5 activity resulted in reduced remyelination. Conditional deletion of CDK5 from olig1+ cells resulted in reduced expression of MBP (25). We found that the expression of CDK5 was decreased by METH, and the expression of CDK5 was restored in TG mice (Figure 4C). Previous studies have revealed that CDK5 is functionally important to timely myelin repair (26) and remyelination occurs after demyelinating events (27). Interestingly, the expression of CDK5 was significantly higher in TG mice than in control mice. Thus, our result suggests that the restored expression of CDK5 in TG mice may play a role in resisting demyelination in spinal cord.

The caspases are cysteine proteases involved in mediating both acute and chronic neuronal cell death (28). The procaspase-12 is localized on the cytoplasmic side of ER and proteolytically activated by excess ER stress and catalyzed by calpain1 (29). ER stress-induced apoptosis has been reported to be mediated by the activation of caspase-12 (10). Caspase-12 deficient mice are resistant to ER stress-induced apoptosis (30). It has been reported ER stress is involved in demyelination (31). The calpain plays a vital role in spinal cord neurodegenerative processes (32, 33). METH induces neurotoxicity through increasing calpain (34). Calpain inhibitor attenuates ER stress-induced cell death in spinal cord (35). MAG is also known as a calpain substrate (36). Calpain-mediated downregulation of MAG has been found in demyelinating diseases, such as multiple sclerosis. Trx-1 resisted neurotoxicity by METH (37). We also certified that Trx-1 suppressed ER stress in Parkinson disease. We found that METH decreased the expressions of procaspase-12 and procaspase-3 and increased the expression of calpain1, whereas overexpression of Trx-1 suppressed these alterations induced by METH treatment (Figures 5A–C). These results suggest that Trx-1 may resist demyelination induced by METH through inhibiting ER stress/calpain1 pathway.

Growing body of evidence suggests that the signaling pathways in the ER stress and inflammation are interconnected through various mechanisms, including activation of the transcription factor NF-κB. NF-κB is a transcription factor and is activated by the outside stimulation, then induces related gene transcription. MAPK cascade acts as an important intracellular mediator of responses closely related to cell growth and differentiation, stress, survival, and cell death (38). The cascade is made up of ERK, JNK, and p38 MAPK. It has been reported that inflammatory factors are induced by ERK (39). METH exposure significantly increases the activity of ERK (40, 41). Several lines of evidence have shown that ERK activation might activate NF-κB (42, 43). Inactivation of both ERK1/2 and NF-κB pathways inhibits the level of TNF-α (44) and inflammation (45, 46). Overproduction of TNF-α and IL-1β results in the injury in spinal cord and finally causes demyelination (47). Agents attenuating TNF-α and/or IL-1β expressions may prevent demyelinating in spinal cord (48). In addition to its antioxidative effects, Trx-1 is known as an anti-inflammatory molecule. Trx-1 resists inflammatory effects induced by METH in spleen (49). Our result also showed that the expressions of NF-κB, IL-1β, and TNF-α, as well as p-ERK level were increased by METH in spinal cord, which were suppressed by Trx-1 overexpression (Figures 6A–D). Thus, our results suggest that Trx-1 may attenuate inflammation induced by METH through blocking ERK/NF-κB signaling pathway.

## Conclusion

The data of this study indicate that METH treatment induces demyelination in spinal cord, whereas Trx-1 protects spinal cord from demyelinating through suppressing ER stress and inflammation. These higher expressions of MAG, MBP, and CDK5 in TG mice increase protecting adaption, thus decrease the sensitivity to METH-induced demyelination. Trx-1 may be a safe and novel therapeutic candidate for demyelination induced by METH in spinal cord.

## Author Contributions

LY, YG, XW, XL, MH, and GC performed the experiments and data analyses. YL did transgenic mice qualification test. LY wrote the manuscript. JB designed the experiments, revised the manuscript, and provided the grants. All the authors agreed with the final manuscript.

## Conflict of Interest Statement

The authors declare that the research was conducted in the absence of any commercial or financial relationships that could be construed as a potential conflict of interest.

## Abbreviations

CDK5, cyclin-dependent kinase 5; CNS, central nervous system; CPP, conditioned place preference paradigm; ER, endoplasmic reticulum; ERK, extracellular signal-regulated kinase; IL-1β, interleukin-1beta; MAG, myelin-associated glycoprotein; MAPK, mitogen-activated protein kinase; MBP, myelin basic protein; METH, methamphetamine; NF-κB, nuclear factor κB; SCI, spinal cord injury; TG, transgenic; TNF-α, tumor necrosis factor-alpha; Trx-1, thioredoxin-1; WT, wild type.

## References

[1] Panenka WJ Procyshyn RM Lecomte T MacEwan GW Flynn SW Honer WG Methamphetamine use: a comprehensive review of molecular, preclinical and clinical findings. Drug Alcohol Depend (2013) 129:167–79. 10.1016/j.drugalcdep.2012.11.016 23273775

[2] Galinato MH Orio L Mandyam CD. Methamphetamine differentially affects BDNF and cell death factors in anatomically defined regions of the hippocampus. Neuroscience (2015) 286:97–108. 10.1016/j.neuroscience.2014.11.042 25463524 PMC4298458

[3] Asser A Taba P Psychostimulants and movement disorders. Front Neurol (2015) 6:75 10.3389/fneur.2015.00075 25941511 PMC4403511

[4] Kuroiwa M Watanabe M Katoh H Suyama K Matsuyama D Imai T Effect of amiloride on endoplasmic reticulum stress response in the injured spinal cord of rats. Eur J Neurosci (2014) 40:3120–7. 10.1111/ejn.12647 24905230

[5] Meng FX Hou JM Sun TS. Effect of oxidative stress induced by intracranial iron overload on central pain after spinal cord injury. J Orthop Surg Res (2017) 12:24. 10.1186/s13018-017-0526-y 28178997 PMC5299723

[6] Wang B Chen C Zhang JT Song RX Yu XC. Triptolide (TPL) improves locomotor function recovery in rats and reduces inflammation after spinal cord injury. Neurol Sci (2015) 36:701–5. 10.1007/s10072-014-2001-4 25547329

[7] He Z Zhou YL Huang Y Wang QQ Zheng BB Zhang HY Dl-3-n-butylphthalide improves functional recovery in rats with spinal cord injury by inhibiting endoplasmic reticulum stress-induced apoptosis. Am J Transl Res (2017) 9:1075–87. 28386335 PMC5376000

[8] Ago T Sadoshima J. Thioredoxin and ventricular remodeling. J Mol Cell Cardiol (2006) 41:762–73. 10.1016/j.yjmcc.2006.08.006 17007870 PMC1852508

[9] Bertini R Howard OM Dong HF Oppenheim JJ Bizzarri C Sergi R Thioredoxin, a redox enzyme released in infection and inflammation, is a unique chemoattractant for neutrophils, monocytes, and T cells. J Exp Med (1999) 189:1783–9. 10.1084/jem.189.11.1783 10359582 PMC2193090

[10] Huh KH Cho Y Kim BS Do JH Park YJ Joo DJ The role of thioredoxin 1 in the mycophenolic acid-induced apoptosis of insulin-producing cells. Cell Death Dis (2013) 4:e721. 10.1038/cddis.2013.247 23846223 PMC3730420

[11] Chae HZ Chung SJ Rhee SG. Thioredoxin-dependent peroxide reductase from yeast. J Biol Chem (1994) 269:27670–8. 7961686

[12] Das KC Das CK. Thioredoxin, a singlet oxygen quencher and hydroxyl radical scavenger: redox independent functions. Biochem Biophys Res Commun (2000) 277:443–7. 10.1006/bbrc.2000.3689 11032742

[13] Lv T Li Y Jia JJ Shi ZZ Bai J. Protective effect of geranylgeranylacetone against methamphetamine-induced neurotoxicity in rat pheochromocytoma cells. Pharmacology (2013) 92:131–7. 10.1159/000353213 24008351

[14] Zeng XS Jia JJ Kwon Y Wang SD Bai J. The role of thioredoxin-1 in suppression of endoplasmic reticulum stress in Parkinson disease. Free Radic Biol Med (2014) 67:10–8. 10.1016/j.freeradbiomed.2013.10.013 24140863

[15] Tzschentke TM. Measuring reward with the conditioned place preference (CPP) paradigm: update of the last decade. Addict Biol (2007) 12:227–462. 10.1111/j.1369-1600.2007.00070.x 17678505

[16] Sabio G Davis RJ. TNF and MAP kinase signalling pathways. Semin Immunol (2014) 26:237–45. 10.1016/j.smim.2014.02.009 24647229 PMC4099309

[17] González B Raineri M Cadet JL García-Rill E Urbano FJ Bisagno V. Modafinil improves methamphetamine-induced object recognition deficits and restores prefrontal cortex ERK signaling in mice. Neuropharmacology (2014) 87:188–97. 10.1016/j.neuropharm.2014.02.002 24530829 PMC5010009

[18] Cao GF Zhu J Zhong Q Shi CF Dang YH Han W Distinct roles of methamphetamine in modulating spatial memory consolidation, retrieval, reconsolidation and the accompanying changes of ERK and CREB activation in hippocampus and prefrontal cortex. Neuropharmacology (2013) 67:144–54. 10.1016/j.neuropharm.2012.10.020 23159329 PMC3582331

[19] Murta V Ferrari C. Peripheral inflammation and demyelinating diseases. Adv Exp Med Biol (2016) 949:263–85. 10.1007/978-3-319-40764-7_13 27714694

[20] Huang MB Kong LP Yang LH Li X Zhou XS Li Y The role of thioredoxin-1 in resisting methamphetamine-induced rewarding effect. Behav Brain Res (2018) 337:280–6. 10.1016/j.bbr.2017.07.047 28782589

[21] Alizadeh A Dyck SM Karimi-Abdolrezaee S. Myelin damage and repair in pathologic CNS: challenges and prospects. Front Mol Neurosci (2015) 8:35. 10.3389/fnmol.2015.00035 26283909 PMC4515562

[22] Wang LC Almazan G. Cdon, a cell surface protein, mediates oligodendrocyte differentiation and myelination. Glia (2016) 64:1021–33. 10.1002/glia.22980 26988125

[23] Harauz G Ishiyama N Hill CM Bates IR Libich DS Farès C. Myelin basic protein-diverse conformational states of an intrinsically unstructured protein and its roles in myelin assembly and multiple sclerosis. Micron (2004) 35:503–42. 10.1016/j.micron.2004.04.005 15219899

[24] Bankston AN Li W Zhang H Ku L Liu G Papa F p39, the primary activator for cyclin-dependent kinase 5 (Cdk5) in oligodendroglia, is essential for oligodendroglia differentiation and myelin repair. J Biol Chem (2013) 288:18047–57. 10.1074/jbc.M113.453688 23645679 PMC3689949

[25] Yang Y Wang HB Zhang J Luo FC Herrup K Bibb JA Cyclin dependent kinase 5 is required for the normal development of oligodendrocytes and myelin formation. Dev Biol (2013) 378:94–106. 10.1016/j.ydbio.2013.03.023 23583582 PMC3686511

[26] Luo FC Zhang J Burke K Miller RH Yang Y. The activators of cyclin-dependent kinase 5 p35 and p39 are essential for oligodendrocyte maturation, process formation, and myelination. J Neurosci (2016) 36:3024–37. 10.1523/JNEUROSCI.2250-15.2016 26961956 PMC4783501

[27] Zhen W Liu A Lu JQ Zhang WD Tattersall D Wang JF. An alternative cuprizone-induced demyelination and remyelination mouse model. ASN Neuro (2017) 9:1759091417725174. 10.1177/1759091417725174 28840755 PMC5574485

[28] Nakagawa T Yuan J. Cross-talk between two cysteine protease families. Activation of caspase-12 by calpain in apoptosis. J Cell Biol (2000) 150:887–94. 10.1083/jcb.150.4.887 10953012 PMC2175271

[29] Takeichi T Wang EL Kitamura O. The effects of low-dose methamphetamine pretreatment on endoplasmic reticulum stress and methamphetamine neurotoxicity in the rat midbrain. Leg Med (2012) 14:69–77. 10.1016/j.legalmed.2011.12.004 22296958

[30] Nakagawa T Zhu H Morishima N Li E Xu J Yankner BA Caspase-12 mediates endoplasmic-reticulum-specific apoptosis and cytotoxicity by amyloid-beta. Nature (2000) 403:98–103. 10.1038/47513 10638761

[31] Ní Fhlathartaigh M McMahon J Reynolds R Connolly D Higgins E Counihan T Calreticulin and other components of endoplasmic reticulum stress in rat and human inflammatory demyelination. Acta Neuropathol Commun (2013) 1:37. 10.1186/2051-5960-1-37 24252779 PMC3893522

[32] Samantaray S Knaryan VH Shields DC Cox AA Haque A Banik NL. Inhibition of calpain activation protects MPTP-induced nigral and spinal cord neurodegeneration, reduces inflammation, and improves gait dynamics in mice. Mol Neurobiol (2015) 52:1054–66. 10.1007/s12035-015-9255-6 26108182 PMC4558265

[33] Samantaray S Knaryan VH Le Gal C Ray SK Banik NL. Calpain inhibition protected spinal cord motoneurons against 1-methyl-4-phenylpyridinium ion and rotenone. Neuroscience (2011) 192:263–74. 10.1016/j.neuroscience.2011.06.007 21723922 PMC3166419

[34] Suwanjang W Phansuwan-Pujito P Govitrapong P Chetsawang B. Calpastatin reduces calpain and caspase activation in methamphetamine-induced toxicity in human neuroblastoma SH-SY5Y cultured cells. Neurosci Lett (2012) 526:49–53. 10.1016/j.neulet.2012.07.066 22897874

[35] Wang C Shi DL Song XH Chen YY Wang LL Zhang XM. Calpain inhibitor attenuates ER stress-induced apoptosis in injured spinal cord after bone mesenchymal stem cells transplantation. Neurochem Int (2016) 97:15–25. 10.1016/j.neuint.2016.04.015 27137651

[36] Vosler PS Brennan CS Chen J. Calpain-mediated signaling mechanisms in neuronal injury and neurodegeneration. Mol Neurobiol (2008) 38:78–100. 10.1007/s12035-008-8036-x 18686046 PMC2726710

[37] Bai J Nakamura H Kwon YW Tanito M Ueda S Tanaka T Does thioredoxin-1 prevent mitochondria- and endoplasmic reticulum-mediated neurotoxicity of 1-methyl-4-phenyl-1,2,3,6-tetrahydropyridine? Antioxid Redox Signal (2007) 9:603–8. 10.1089/ars.2006.1513 17465883

[38] Johnson GL Lapadat R. Mitogen-activated protein kinase pathways mediated by ERK, JNK, and p38 protein kinases. Science (2002) 298:1911–2. 10.1126/science.1072682 12471242

[39] Xu M Wang HF Zhang YY Zhuang HW. Protection of rats spinal cord ischemia-reperfusion injury by inhibition of MiR-497 on inflammation and apoptosis: possible role in pediatrics. Biomed Pharmacother (2016) 81:337–44. 10.1016/j.biopha.2016.04.028 27261611

[40] Son JS Jeong YC Kwon YB. Regulatory effect of bee venom on methamphetamine-induced cellular activities in prefrontal cortex and nucleus accumbens in mice. Biol Pharm Bull (2015) 38:48–52. 10.1248/bpb.b14-00539 25744457

[41] Sun LL Zhang Y Liu JF Wang J Zhu WL Zhao LY Role of melanin-concentrating hormone in the nucleus accumbens shell in rats behaviourally sensitized to methamphetamine. Int J Neuropsychopharmacol (2013) 16:1767–80. 10.1017/S1461145713000072 23449013

[42] Ou Z Tao MX Gao Q Zhang XL Yang Y Zhou JS Up-regulation of angiotensin-converting enzyme in response to acute ischemic stroke via ERK/NF-kappaB pathway in spontaneously hypertensive rats. Oncotarget (2017) 8:97041–51. 10.18632/oncotarget.21156 29228591 PMC5722543

[43] Dhawan P Singh AB Ellis DL Richmond A. Constitutive activation of Akt/protein kinase B in melanoma leads to up-regulation of nuclear factor-kappaB and tumor progression. Cancer Res (2002) 62:7335–42. 12499277

[44] Seo H Cho YC Ju A Lee S Park BC Park SG Dual-specificity phosphatase 5 acts as an anti-inflammatory regulator by inhibiting the ERK and NF-kappaB signaling pathways. Sci Rep (2017) 7:17348 10.1038/s41598-017-17591-9 29229953 PMC5725455

[45] Choi KC Hwang JM Bang SJ Kim BT Kim DH Chae M Chloroform extract of alfalfa (*Medicago sativa*) inhibits lipopolysaccharide-induced inflammation by downregulating ERK/NF-kappaB signaling and cytokine production. J Med Food (2013) 16:410–20. 10.1089/jmf.2012.2679 23631491

[46] Park JH Seo YH Jang JH Jeong CH Lee S Park B. Asiatic acid attenuates methamphetamine-induced neuroinflammation and neurotoxicity through blocking of NF-kB/STAT3/ERK and mitochondria-mediated apoptosis pathway. J Neuroinflammation (2017) 14:240. 10.1186/s12974-017-1009-0 29228978 PMC5725763

[47] Garraway SM Woller SA Huie JR Hartman JJ Hook MA Miranda RC Peripheral noxious stimulation reduces withdrawal threshold to mechanical stimuli after spinal cord injury: role of tumor necrosis factor alpha and apoptosis. Pain (2014) 155:2344–59. 10.1016/j.pain.2014.08.034 25180012 PMC4253555

[48] Choi JH Lee MJ Jang M Kim EJ Shim I Kim HJ An oriental medicine, hyungbangpaedok-san attenuates motor paralysis in an experimental model of multiple sclerosis by regulating the T cell response. PLoS One (2015) 10:e0138592 10.1371/journal.pone.0138592 26444423 PMC4596626

[49] Wu XL Li X Li Y Kong LP Fang JL Zhou XS The overexpression of thioredoxin-1 suppressing inflammation induced by methamphetamine in spleen. Drug Alcohol Depend (2016) 159:66–71. 10.1016/j.drugalcdep.2015.11.021 26684867

